# A Multimodule Collaborative Framework for Unsupervised Visible–Infrared Person Re-Identification with Channel Enhancement Modality

**DOI:** 10.3390/s26061770

**Published:** 2026-03-11

**Authors:** Baoshan Sun, Yi Du, Liqing Gao

**Affiliations:** 1 School of Computer Science and Technology, Tiangong University, Tianjin 300387, China; sunbaoshan@tiangong.edu.cn (B.S.); 2431101245@tiangong.edu.cn (Y.D.); 2 Tianjin Key Laboratory of Autonomous Intelligence Technology and Systems, Tiangong University, Tianjin 300387, China

**Keywords:** unsupervised learning, cross-modality, person re-identification, feature alignment, channel enhancement

## Abstract

Unsupervised visible–infrared person re-identification (USL-VI-ReID) plays a pivotal role in cross-modal computer vision applications for intelligent surveillance and public safety. However, the task remains hampered by large modality gaps and limited granularity in feature representations. In particular, channel augmentation (CA) is typically used only for data augmentation, and its potential as an independent input modality remains unexplored. To address these shortcomings, we present a multimodule collaborative USL-VI-ReID framework that explicitly treats CA as a separate input modality. The framework combines four complementary modules. The Person-ReID Adaptive Convolutional Block Attention Module (PA-CBAM) module extracts discriminative features using a two-level attention mechanism that refines salient spatial and channel cues. The Varied Regional Alignment (VRA) module performs cross-modal regional alignment and leverages the Multimodal Assisted Adversarial Learning (MAAL) to reinforce region-level correspondence. The Varied Regional Neighbor Learning (VRNL) implements reliable neighborhood learning via multi-region association to stabilize pseudo-labels and capture local structure. Finally, the Uniform Merging (UM) module merges split clusters through alternating contrastive learning to improve cluster consistency. We evaluate the proposed method on SYSU-MM01 and RegDB. On RegDB’s visible-to-infrared setting, the approach achieves Rank-1 = 93.34%, mean Average Precision (mAP) = 87.55%, and mean Inverse Negative Penalty (mINP) = 76.08%. These results indicate that our method effectively reduces modal discrepancies and increases feature discriminability. It outperforms most existing unsupervised baselines and several supervised approaches, thereby advancing the practical applicability of USL-VI-ReID.

## 1. Introduction

Person re-identification (ReID) aims to accurately match images of the same pedestrian captured from different camera angles and environmental conditions, providing essential support for intelligent surveillance and public safety [1,2,3,4]. As surveillance systems evolve to meet all weather operational demands, infrared (IR) functionality in devices is gaining popularity. This technology effectively addresses the challenges of blurred visible light imaging in nighttime or low-light conditions. Given the higher incidence of security incidents at night, visible–infrared person re-identification (VI-ReID) has emerged as a key research area in cross-modal computer vision. This focus aims to enable effective matching between visible light and infrared images [5,6,7,8].

While existing VI-ReID research has progressed [9,10,11,12], many methods achieve high recognition accuracy using complex feature alignment strategies. However, these methods often depend on large-scale labeled cross-modal datasets [6,13,14,15]. Manually labeling such data is resource-intensive and fails to adequately represent complex, dynamic real-world scenarios, limiting industrial applications. In real-world intelligent surveillance, unsupervised methods play a crucial role in visible–infrared person re-identification (VI-ReID) when annotation resources are limited. Surveillance systems continuously produce vast amounts of cross-modal visual data (visible and infrared), making it infeasible to label every pedestrian image because of the substantial time and labor required. Moreover, surveillance environments change frequently—pedestrian flow, lighting, and camera placements all vary—so labeled datasets collected in one scene often fail to generalize to new, unlabeled deployments. By contrast, unsupervised VI-ReID directly learns discriminative cross-modal features from unlabeled data, eliminates heavy dependence on manual annotation, and adapts to the evolving characteristics of operational surveillance. These properties make unsupervised VI-ReID essential for deploying VI-ReID technology in practical intelligent surveillance systems. To address this, unsupervised visible–infrared person re-identification (USL-VI-ReID) has emerged. This approach generates pseudo-identity labels through clustering, reducing the need for manual annotation, and is now a key focus in VI-ReID research [16,17,18,19,20].

Despite these advancements and the clear practical demand, existing USL-VI-ReID methods still suffer from three critical limitations that hinder their performance, which our framework is specifically designed to address:

- Imperfect Cross-Modal Alignment: Visible light and infrared images differ fundamentally in their imaging principles, leading to significant disparities in their feature distributions that are difficult to reconcile.
- Insufficient Local Feature Representation: Most existing studies rely on a single global feature for matching, which struggles to capture fine-grained discriminative details (e.g., clothing textures and body parts). This approach is vulnerable to pose changes, occlusions, and illumination fluctuations, reducing the reliability of cross-modal similarity calculations and ultimately decreasing recognition accuracy [18,19,21].
- Underutilization of Channel Augmentation (CA): Traditional methods treat CA merely as a simple data augmentation tool, overlooking its potential as an independent input modality. They fail to leverage CA images effectively to bridge the gap between visible light and infrared modalities, further exacerbating the inconsistency in feature distributions between these modalities.

**Figure 1:**
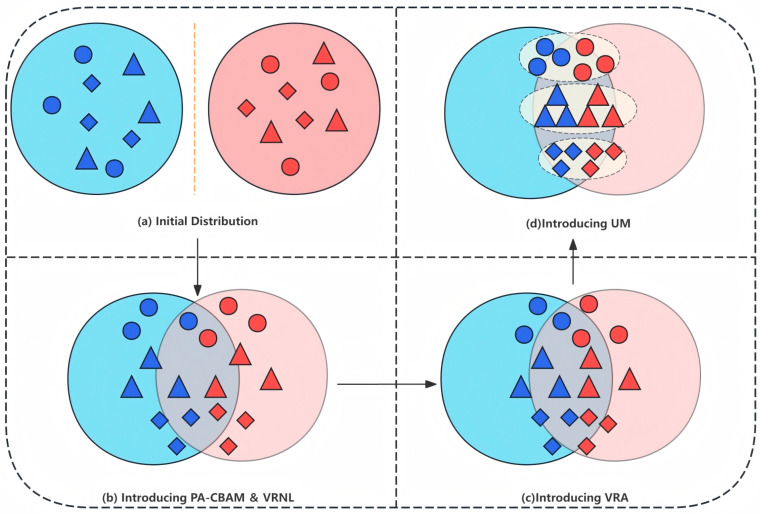
Conceptual Overview of the proposed USL-VI-ReID framework, illustrating the core design of treating Channel Augmentation (CA) as an independent input modality (instead of mere data augmentation) and the collaborative workflow of its four key modules (PA-CBAM, VRA, VRNL, UM). CA serves as a critical bridge modality between visible and infrared spectra, providing complementary feature information to mitigate the inherent modal gap and support cross-modal alignment for the subsequent modules. The framework sequentially addresses the core challenges in unsupervised visible–infrared person re-identification: large cross-modal gaps, insufficient fine-grained feature representation, and low utilization of channel enhancement information.

To comprehensively tackle the three aforementioned limitations, this paper introduces the USL-VI-ReID framework, which integrates multimodule collaborative optimization. As depicted in Figure 1, Channel Augmentation (CA) is treated as input data rather than just a data augmentation technique. By employing refined feature extraction, cross-modal regional alignment, and clustering integration, the framework enhances the discriminability and consistency of cross-modal pedestrian features. The framework comprises four core modules: First, the Person-ReID Adaptive Convolutional Block Attention Module (PA-CBAM) combines channel attention with multi-scale spatial attention to boost the channel discriminability of local pedestrian identity features and accurately capture key region information across scales, as shown in Figure 1b. Second, the Varied Regional Alignment (VRA) module incorporates the Multimodal Assisted Adversarial Learning (MAAL) module. This assists in identifying and matching diverse regional features across different modalities, utilizing channel augmentation technology to minimize modal differences and facilitate cross-modal association, as shown in Figure 1c. Third, the Varied Regional Neighbor Learning (VRNL) module ensures reliable neighbor learning through multi-region spatial association, as shown in Figure 1b. Fourth, the Uniform Merging (UM) module consolidates split clusters of the same ID, suppresses noise interference, and enhances the robustness of feature representation, as shown in Figure 1d.

The main contributions of this paper can be summarized into the following four points, which directly address the critical limitations of previous methods in local feature representation, cross-modal alignment, and channel augmentation utilization:

- The PA-CBAM module is designed to tackle the limitation of insufficient local feature representation. It uses a two-stage attention strategy—first optimizing channel responses, then refining spatial activations—to strengthen the discriminative representation of pedestrian identities. By producing more granular and locally informative features, PA-CBAM creates a robust foundation for subsequent cross-modal alignment and neighborhood learning and remedy shortcomings of traditional single global descriptors.
- The VRA module is proposed to achieve precise cross-modal alignment while fully exploiting the potential of channel augmentation. It incorporates the MAAL component and applies channel enhancement techniques to reduce modal discrepancies and enable cross-modal regional feature matching. In doing so, VRA moves beyond conventional channel enhancement approaches that serve merely as data augmentation and thus cannot fully bridge the modal gap.
- The VRNL module combines multi-region spatial association with multimodal adversarial learning and imposes parameter constraints to prevent excessive cross-modal matching. This design improves the reliability of neighborhood learning and enforces modal consistency, addressing the tendency of neighborhood learning to suffer from modal differences and the risk that overmatching will induce identity confusion.
- The UM module performs alternating contrastive learning to merge split clusters that correspond to the same identity, thereby reducing intra-modal variance and increasing robustness to noise. This mechanism counters problems encountered during preprocessing, including identity clustering fragmentation, large within-modal feature disparities, and interference from noisy samples.

## 2. Related Work

This section examines key research directions in visible–infrared person re-identification. It reviews the current state of supervised visible–infrared re-identification, unsupervised single-modality re-identification, and unsupervised visible–infrared re-identification. The analysis highlights the strengths and limitations of existing methods, providing a foundation for the proposed approach in this paper.

### 2.1. Supervised Visible–Infrared Person Re-Identification

Supervised visible–infrared person re-identification (SVI-ReID) depends on extensive annotated cross-modal datasets. The primary goal is to bridge the inherent gap between visible and infrared modalities through an effective feature learning mechanism, ensuring precise cross-modal matching. Two main technical approaches have emerged: subspace learning and generative transformation. Subspace learning methods aim to create a shared feature space across modalities and enhance discriminability by adjusting feature distribution. MCLNet [22] employs a multi-branch network to emphasize identity-related features within this common subspace. DART enhances the feature subspace using a dynamic attention mechanism, significantly improving cross-modal feature consistency. Furthermore, MAUM [23] introduces a multi-scale alignment module to reduce the modality gap, showcasing strong feature alignment capabilities. FMCNet [24] employs a generative network to create cross-modal intermediate features. Despite their benefits, generative methods often incur high computational costs and may introduce image noise. For instance, GAN-based methods [25,26,27,28,29,30] can convert modalities but struggle to balance real-time performance with feature purity, limiting their industrial application.

The SVI-ReID method has achieved high recognition accuracy; however, its reliance on manual annotation limits its effectiveness in complex and dynamic real-world scenarios. The costs associated with annotation and the method’s adaptability to various scenes have become significant bottlenecks in advancing this research area.

### 2.2. Unsupervised Single-Modal Person Re-Identification

Unsupervised single-modal person re-identification (USL-ReID) focuses on extracting distinct features from unlabeled single-modal data, primarily visible light. The main approach involves generating pseudo-labels via clustering and employing contrastive learning strategies to enhance feature representation. This method significantly minimizes reliance on labeled data. Clustering and pseudo-label optimization are central to USL-ReID. Cluster-Contrast [20] creates a cluster-level memory dictionary and employs a momentum update strategy to reduce noisy pseudo-label propagation. ICE [31] enhances feature discrimination through inter-instance contrastive coding and pairwise similarity scores. Additionally, CAP [3] introduces a novel approach by dividing each cluster into multiple camera proxies. This method addresses intra-class differences for the same identity, offering a fresh perspective on improving pseudo-label quality. The advancement of contrastive learning strategies has significantly propelled the development of USL-ReID. For instance, IICS [32] improves local features using cross-image patch contrastive learning, while PPLR [33] introduces a progressive pseudo-label refinement mechanism to enhance feature discriminability. When applied to cross-modal scenarios, they fail to address the inherent feature distribution disparities between visible and infrared images. This oversight results in limited modal adaptability and a notable drop in performance.

### 2.3. Unsupervised Visible–Infrared Person Re-Identification

Unsupervised visible–infrared person re-identification (USL-VI-ReID) is a prominent research area. It aims to accurately match visible and infrared images in scenarios involving unlabeled cross-modal data. This is achieved through modal difference suppression, pseudo-label generation, and cross-modal association construction. The research primarily focuses on two areas: modal alignment and cross-modal clustering association.

Modal difference suppression often employs multimodal adversarial learning and feature enhancement techniques. ADCA [34] introduces a dual contrastive learning framework that narrows the modal gap by aggregating instances and memory features. Similarly, PGMAL [18] incorporates a graph matching mechanism with adversarial learning to optimize cross-modal feature distribution, enhancing feature consistency. Despite these advancements, many adversarial learning methods primarily target global feature alignment and fall short in addressing discrepancies in local key features, such as clothing textures and body part details. This limitation reduces the reliability of cross-modal similarity calculations. Developing cross-modal clustering associations is a key focus in USL-VI-ReID. Techniques like OTLA [19] and DOTLA [16] employ the optimal transport algorithm for cross-modal clustering matching. Meanwhile, MBCCM [35] enhances label assignment using multi-branch clustering associations. However, many of these approaches match clusters directly without adequate modal alignment, leading to potential errors in label assignment due to modal bias. This issue becomes more pronounced when there is an imbalance in the number of visible light and infrared clusters, significantly increasing the error rate.

Another representative multimodule collaborative approach is ReID-DeePNet [36], which addresses background clutter interference through a hybrid deep learning framework: it first fuses Mask R-CNN and GrabCut for precise pedestrian segmentation to suppress background noise, then extracts discriminative features via dual models (CNN and Deep Belief Network, DBN) and fuses their matching scores to improve re-identification accuracy. This work highlights the effectiveness of “background suppression + multi-model feature fusion” in enhancing feature discriminability, which aligns with our framework’s design philosophy of leveraging multimodule synergy (e.g., PA-CBAM for feature refinement, VRA for cross-modal alignment) to mitigate modal gaps and background interference. Notably, ReID-DeePNet verifies that integrating specialized preprocessing modules (e.g., segmentation) with multi-model feature learning can significantly improve ReID performance, providing valuable insights for our adoption of CA as an independent modality to bridge cross-modal feature discrepancies.

Existing methods often struggle with insufficient granularity in feature representation. Typically, they depend on a single global feature for matching, which fails to capture local key details of pedestrians and is susceptible to pose changes, occlusions, and lighting variations. Additionally, channel augmentation (CA) technology is primarily used for data augmentation. Its potential as an independent input modality to bridge modality gaps remains underexplored. This oversight exacerbates feature distribution inconsistencies between modalities, limiting performance improvements in USL-VI-ReID.

## 3. Methods

**Figure 2:**
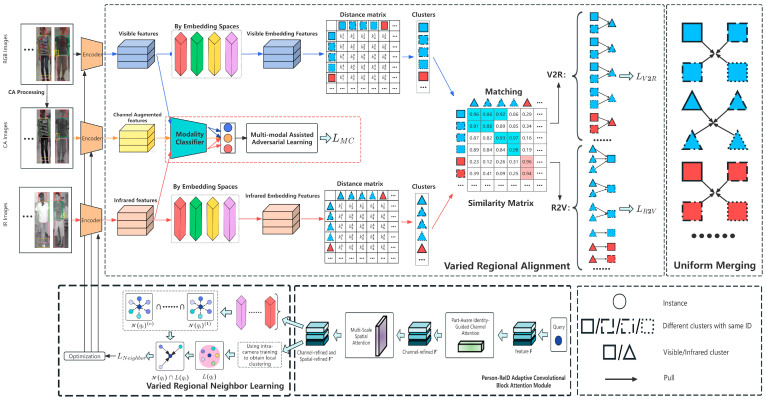
Illustration of the pipeline of our proposed multimodule collaborative USL-VI-ReID model, which integrates Channel Augmentation (CA) as an independent input modality (rather than a simple data augmentation tool) alongside the Person-ReID Adaptive Convolutional Block Attention Module (PA-CBAM), Varied Regional Alignment (VRA), Varied Regional Neighbor Learning (VRNL), and Uniform Merging (UM). As a stand-alone modality, CA provides complementary cross-spectral feature cues to bridge the visible–infrared modal gap and collaborates with the four core modules throughout the entire progressive optimization process of the model. The model realizes step-by-step performance optimization through refined feature extraction, cross-modal difference reduction, regional feature alignment, and final clustering merging, with all modules working synergistically to enhance the discriminability and consistency of cross-modal pedestrian features for high-performance USL-VI-ReID.

The framework of the proposed method is illustrated in Figure 2. Initially, we employ the Varied Regional Neighbor Learning (VRNL) module to harness associations among multiple regional spaces, enhancing neighbor learning reliability. Concurrently, the PA-CBAM module is integrated into VRNL for more precise feature extraction. Building on VRNL, we introduce the Varied Regional Alignment (VRA) module, designed to effectively identify and match diverse regional features across different modalities. The MAAL module supports the VRA module by utilizing channel enhancement technology to reduce modal differences, aiding subsequent cross-modal associations. Lastly, the Uniform Merging (UM) module integrates different clusters with the same identifier (ID) identified and matched by VRNL. This integration aims to reduce intra-modal gaps, minimize noise impact, and enhance the robustness and accuracy of feature representation.

### 3.1. The Baseline Re-ID Mode

In USL-VI-ReID, the training data includes visible light data $X^{v}=\left\{x_{1}^{v},x_{2}^{v},\ldots ,x_{N^{v}}^{v}\right\}$ and infrared data $X^{r}=\left\{x_{1}^{r},x_{2}^{r},\ldots ,x_{N^{r}}^{r}\right\}$, represented as $N^{v}$ and $N^{r}$, respectively, indicating their sample sizes. Existing studies [18,34,37] highlight that channel augmentation (CA) images significantly help bridge the gap between visible and infrared modalities. Consequently, we incorporated CA modality images into the input data to enhance the generalization ability of the visible modality. The CA data is represented as $X^{CA}=\left\{x_{1}^{CA},x_{2}^{CA},\ldots ,x_{N^{v}}^{CA}\right\}$.

We adopt the momentum update strategy of Yang [1] to refresh the memories for all three modalities.

At the start of each training round, we build modality-specific clustering memory using the following formula:

(1) $$\Phi_{k}^{m}=\frac{1}{\left|H_{k}^{m}\right|}\underset{u_{n}^{m}\in H_{k}^{m}}{\sum }u_{n}^{m}$$

In this context, $\left|H_{k}^{m}\right|$ denotes the number of instances in the k-th clustering set, while $u_{n}^{m}$ also refers to the instance features concatenated with each Regional Space. The variable m can assume three values: {v, r, c}, corresponding to the visible light modality, the infrared modality, and the CA modality, respectively.

In this paper, the Vision Transformer network extracts query features from visible light, infrared, and CA data, denoted as $F^{v}$, $F^{r}$, and $F^{c}$, respectively.

For the loss function, using the visible and infrared query features $F^{v}$ and $F^{r}$, the contrastive loss is calculated with the following formula:

(2) $$L_{\mathrm{id}}^{v}=-\log\frac{\exp\left(F^{v}\cdot \varphi_{+}^{v}/\tau \right)}{\sum_{k=0}^{N^{v}}\exp\left(F^{v}\cdot \varphi_{k}^{v}/\tau \right)}$$

(3) $$L_{\mathrm{id}}^{r}=-\log\frac{\exp\left(F^{r}\cdot \varphi_{+}^{r}/\tau \right)}{\sum_{k=0}^{N^{r}}\exp\left(F^{r}\cdot \varphi_{k}^{r}/\tau \right)}$$

(4) $$L_{\mathrm{id}}=L_{\mathrm{id}}^{v}+L_{\mathrm{id}}^{r}$$

In this context, $\varphi_{+}^{v}$ and $\varphi_{+}^{r}$ represent the positive clustering for visible-light and infrared memory, respectively, corresponding to the pseudo-label of the query feature F. $\varphi_{k}^{v}$ represents the features of the k-th cluster in the visible light modality clustering memory and $\varphi_{k}^{r}$ represents the features of the k-th cluster in the infrared light modality clustering memory. Additionally, τ denotes the temperature coefficient.

### 3.2. Person-ReID Adaptive Convolutional Block Attention Module

In feature extraction, we utilize the CBAM module concept [38] to develop the Person-ReID Adaptive CBAM (PA-CBAM) module. This module comprises two enhancement sub-modules: Part-Aware Identity-Guided Channel Attention (PI-CA) and Multi-Scale Spatial Attention (M-SA). Initially, PI-CA optimizes the input feature map in the channel dimension. Subsequently, M-SA refines it in the spatial dimension, resulting in a highly discriminative feature map.

**Figure 3:**
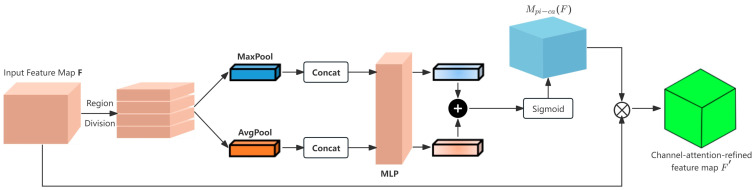
Architecture of the Part-Aware Identity-Guided Channel Attention (PI-CA) module. The module takes a pedestrian feature map $F\in R^{C\times H\times W}$ (C: channel number, H: height, W: width) as input, first splits it into K horizontal regional blocks, then performs average/max pooling on each block to generate part-level descriptors $F_{c,\mathrm{local}}^{\mathrm{avg}},F_{c,\mathrm{local}}^{\max}\in R^{C\times 1\times K}$, and finally outputs a channel attention-refined feature map $F^{\prime }\in R^{C\times H\times W}$ with the same spatial dimension as the input after identity-guided MLP and sigmoid activation.

PI-CA strengthens the model’s ability to discern local features and heightens its attention to identity-related characteristics, as shown in Figure 3. We segment the input feature map $F\in R^{C\times H\times K}$ into K distinct horizontal region blocks $\left\{F_{1},F_{2},\ldots ,F_{K}\right\}$. For each block, we apply average pooling and max pooling to retain the spatial context of each segment, producing two sets of part-level descriptors:

(5) $$F_{c,\mathrm{local}}^{\mathrm{avg}}=\mathrm{Concat}\left(\mathrm{AvgPool}\left(F_{1}\right),\mathrm{AvgPool}\left(F_{2}\right),\ldots ,\mathrm{AvgPool}\left(F_{k}\right)\right)\in R^{C\times 1\times K}$$

(6) $$F_{c,\mathrm{local}}^{\max}=\mathrm{Concat}\left(\mathrm{MaxPool}\left(F_{1}\right),\mathrm{MaxPool}\left(F_{2}\right),\ldots ,\mathrm{MaxPool}\left(F_{k}\right)\right)\in R^{C\times 1\times K}$$

In this context, Concat(·) refers to the concatenation operation along the channel dimension. This operation integrates part-level contextual information into a global descriptor, maintaining the distinctiveness of each identity part.

This paper presents an identity-guided regularization term in the multi-layer perceptron (MLP) of channel attention to suppress background-related channels and enhance identity-related ones. This regularization uses identity pseudo-labels to maintain consistent channel weights for samples with the same identity while increasing differences in channel weights among samples with different identities. The modified MLP calculation formula incorporating this regularization is as follows:

(7) $$\mathrm{MLP}\left(X\right)=W_{1}\left(\mathrm{ReLU}\left(W_{0}X\right)+\lambda \cdot R\left(W_{0}X,y\right)\right)$$

In this context, $X\in \left\{F_{c,\mathrm{local}}^{\mathrm{avg}},F_{c,\mathrm{local}}^{\max}\right\}$ denotes the part-level descriptor. The weight parameters of the MLP are represented by $W_{0}\in R^{C/r\times C}$ and $W_{1}\in R^{C\times C/r}$, with r serving as the dimensionality reduction coefficient. The sample’s identity pseudo-label is indicated by y. Additionally, $R\left(\cdot \right)$ refers to the identity consistency regularization term, while λ stands for the regularization weight. Specifically, λ is used to adjust the strength of the identity-guided regularization constraint: a larger λ enhances the suppression of background-related channels and the emphasis on identity-related channels, while an overly large λ may lead to over-fitting of pseudo-labels. A moderate λ ensures the module refines identity-discriminative features without being affected by noisy pseudo-labels.

The channel attention map $M_{\mathrm{pi}-\mathrm{ca}}\left(F\right)$ is calculated by integrating part-level descriptors with the identity-guided MLP. The formula is as follows:

(8) $$M_{\mathrm{pi}-\mathrm{ca}}\left(F\right)=\sigma \left(\mathrm{MLP}\left(F_{c,\mathrm{local}}^{\mathrm{avg}}\right)+\mathrm{MLP}\left(F_{c,\mathrm{local}}^{\max}\right)\right)$$

Among these, $\sigma \left(\cdot \right)$ represents the sigmoid function, which normalizes channel weights to the [0, 1] interval. The feature map, enhanced by channel attention, is derived through element-wise multiplication:

(9) $$F^{\prime }=M_{\mathrm{pi}-\mathrm{ca}}\left(F\right)⊗F$$

**Figure 4:**
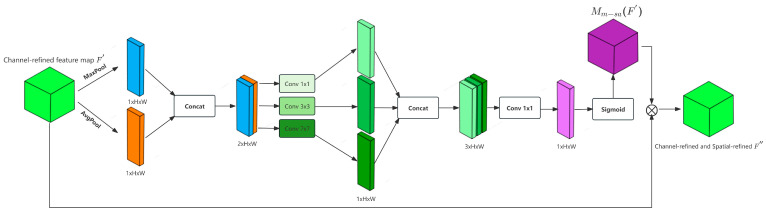
Architecture of the Multi-Scale Spatial Attention (M-SA) module. The module takes the channel-refined feature map $F^{\prime }\in R^{C\times H\times W}$ (output of PI-CA) as input, first aggregates channel information via average/max pooling to get a concatenated descriptor $F_{s,\mathrm{cat}}\in R^{2\times H\times W}$, then extracts multi-scale spatial features through 1 × 1, 3 × 3 and 7 × 7 convolutions to generate $F_{s,\mathrm{multi}}\in R^{3\times H\times W}$, and finally outputs a spatial-and-channel dual-refined feature map $F^{\prime \prime }\in R^{C\times H\times W}$ after 1 × 1 convolution fusion and sigmoid spatial attention weighting.

M-SA introduces a multi-scale convolution block to tackle the challenge of varying sizes of discriminative regions in person re-identification tasks, as shown in Figure 4. This block incorporates three convolution kernel sizes: 1 × 1, 3 × 3, and 7× 7. The 1 × 1 kernel captures global spatial dependencies, the 3 × 3 kernel targets local regions, and the 7 × 7 kernel models large-scale structural features. Initially, for the input feature map $F^{\prime }$ optimized by PI-CA, average and max pooling are applied along the channel dimension to aggregate channel information.

(10) $$F_{s,\mathrm{cat}}=\mathrm{Concat}\left(\mathrm{AvgPool}{\left(F^{\prime }\right)}_{\mathrm{channel}},\mathrm{MaxPool}{\left(F^{\prime }\right)}_{\mathrm{channel}}\right)\in R^{2\times H\times W}$$

Among these operations, $\mathrm{AvgPool}{\left(F^{\prime }\right)}_{\mathrm{channel}}$ denotes average pooling, and $\mathrm{MaxPool}{\left(F^{\prime }\right)}_{\mathrm{channel}}$ signifies maximum pooling along the channel axis. The concatenated descriptor $F_{s,\mathrm{cat}}$ is then fed into the multi-scale convolution block.

(11) $$F_{s,\mathrm{multi}}=\mathrm{Concat}\left(f_{1\times 1}\left(F_{s,\mathrm{cat}}\right),f_{3\times 3}\left(F_{s,\mathrm{cat}}\right),f_{7\times 7}\left(F_{s,\mathrm{cat}}\right)\right)\in R^{3\times H\times W}$$

In this context, $f_{k\times k}\left(\cdot \right)$ denotes a convolution operation with a kernel size of k × k. Following this, the multi-scale features are combined into a single-channel feature map using a 1 × 1 convolution.

(12) $$F_{s,\mathrm{fusion}}=f_{1\times 1}\left(F_{s,\mathrm{multi}}\right)\in R^{1\times H\times W}$$

The spatial attention map $M_{\mathrm{mp}-\mathrm{sa}}\left(F^{\prime }\right)$ is obtained through the sigmoid function:

(13) $$M_{\mathrm{mp}-\mathrm{sa}}\left(F^{\prime }\right)=\sigma \left(F_{s,\mathrm{fusion}}\right)$$

The final feature map optimized by PA-CBAM is as follows:

(14) $$F^{\prime \prime }=M_{\mathrm{mp}-\mathrm{sa}}\left(F^{\prime }\right)⊗F^{\prime }$$

### 3.3. Varied Regional Alignment

Current research methods often use channel enhancement techniques to improve intra-modal learning and clustering in visible light images. However, these approaches frequently neglect the alignment of distributions among channel-enhanced, visible light, and infrared images, treating enhancement merely as data augmentation. Inspired by MCLNet [22], CmGAN [39], and PCAL [40], we propose Multimodal Assisted Adversarial Learning (MAAL). MAAL aims to optimize the use of each modality, bridging the feature distribution gap across different modalities, thereby enhancing the model’s adaptability to cross-modal data and improving feature alignment. By employing an adversarial learning strategy, we leverage data from all three modalities to boost the model’s generalization, minimize modal discrepancies, and support model training. The modal classification loss is defined as follows:

(15) $$L_{m}\left(E\right)=\underset{m\in \left\{v,r,c\right\}}{\sum }\mathrm{CE}\left(W_{c}\left(F_{m}\right){,t}_{m}\right)$$

Among these, $F_{m}$ can assume values of $F_{v}$, $F_{r}$, and $F_{c}$, representing features of visible light, infrared, and channel-enhanced query samples, respectively. Similarly, $t_{m}$ can take values of $t_{v}$, $t_{r}$, and $t_{c}$, indicating labels for the three modalities: visible light, infrared, and channel enhancement. The symbol $W_{c}$ denotes the modality classifier, while E signifies its parameters. CE stands for cross-entropy loss.

Building on this foundation, we introduced an additional modal classification loss. This loss aims to categorize the features of all three modalities into the channel-enhanced modality. The formula is as follows:

(16) $$L_{c}\left(\theta \right)=\mathrm{CE}\left(W_{c}\left(F_{v,r,c}\right),t_{c}\right)$$

In this context, $F_{v,r,c}$ denotes the query sample features across three modalities: visible light, infrared, and channel enhancement. Additionally, θ signifies the parameters of the backbone network.

The total loss in the three-modal adversarial learning module can be expressed as

(17) $$L_{mc}=\alpha L_{m}+\beta L_{c}$$

Here, α and β are the weight hyperparameters of the modal classification loss $L_{m}$ and the channel-augmented modal classification loss $L_{c}$, respectively. α regulates the intensity of the modality discrimination constraint for the three modalities (visible, infrared, CA), ensuring the model can distinguish different modal features; β controls the strength of the constraint that maps all modal features to the CA modal space, which is the core to realize cross-modal feature alignment with CA as the bridge modality. The collaborative adjustment of α and β balances the dual objectives of “modal discrimination” and “cross-modal alignment” in adversarial learning. An alternating optimization approach is employed: the modality classifier is optimized using $L_{m}$, while the backbone network is optimized using $L_{c}$. Treating channel enhancement as an additional modality and integrating it into the training process ensures better distribution alignment of features across the three modalities—visible light, infrared, and channel enhancement—within the same probability space. This method is more effective than using channel enhancement solely for data augmentation.

Next, in the regional space, we calculated the distances between various features of visible light and infrared. We then constructed a distance matrix for these two modalities and clustered the features based on this matrix.

After obtaining the clusters for the visible light and infrared modalities, we denote the similarity matrix as $S_{total}=\left\{S\left(i,j\right)\right\}.$ The calculation method for this similarity matrix is as follows:

(18) $$S\left(i,j\right)=\overset{N}{\underset{n=1}{\sum }}\exp\left(\mathrm{sim}\left(\varphi_{i}^{v\left(n\right)},\varphi_{j}^{r\left(n\right)}\right)\right)$$

(19) $$\mathrm{sim}\left(\varphi_{i}^{v\left(n\right)},\varphi_{j}^{r\left(n\right)}\right)=\frac{\varphi_{i}^{v\left(n\right)}\cdot \varphi_{j}^{r\left(n\right)}}{\left|\varphi_{i}^{v\left(n\right)}\left|\times \right|\varphi_{j}^{r\left(n\right)}\right|}$$

(20) $$\varphi_{i}^{v\left(n\right)}=\frac{1}{\left|H_{k}^{v}\right|}\underset{u_{L}^{v}\in H_{k}^{v}}{\sum }u_{L}^{v\left(n\right)},n=1,\ldots ,N$$

(21) $$\varphi_{i}^{r\left(n\right)}=\frac{1}{\left|H_{k}^{r}\right|}\underset{u_{L}^{r}\in H_{k}^{r}}{\sum }u_{L}^{r\left(n\right)},n=1,\ldots ,N$$

In this context, S(i,j) denotes the similarity within cluster $\varphi_{i}^{v\left(n\right)}$ and $\varphi_{i}^{r\left(n\right)}$, while n indicates the nth regional space. Additionally, $u_{L}^{v\left(n\right)}$ represents the Lth instance feature within this regional space, with the same notation applying to $u_{L}^{r\left(n\right)}$. Furthermore, $\left|H_{k}^{v}\right|$ signifies the number of instances in the kth cluster $H_{k}^{v}$ within the visible light modality, and this notation is consistent for $\left|H_{k}^{r}\right|$.

After obtaining the similarity matrix, we aim to ensure that clusters with the same ID maintain consistent cross-modal labels. In each cycle, we select the pair of cross-modal clusters with the highest similarity from this matrix. The screening formula is as follows:

(22) $$\left(i,j\right)=\arg\underset{i,j}{\max}\;S_{\mathrm{total}},i=1,\ldots ,N^{v},j=1,\ldots ,N^{r}$$

Arg max identifies the row and column numbers, i and j, where the maximum value in the similarity matrix occurs. These indices correspond to the pair of clusters with the highest similarity.

To ensure stability during matching and reduce computational demands, we set a parameter, μ, to limit the number of matches between modalities. This helps prevent excessive matching and identity confusion. The parameter μ can restrict matches for either visible light or infrared clusters. In this study, we applied μ specifically to limit matches for each infrared cluster.

We employed the TokenMatcher method [37] to learn cross-modal features through modal shared contrast. This process involves two components: visible-to-infrared modal learning and infrared-to-visible modal learning. The corresponding loss functions are expressed as follows:

(23) $$\varphi_{k}^{\mathrm{vs}}=\frac{\left(\sum_{u_{n}^{v}\in H_{k}^{v}}u_{n}^{v}+\sum_{u_{n}^{r}\in H_{V2R\left[k\right]}^{r}}u_{n}^{r}\right)}{\left|H_{k}^{v}\cup H_{V2R\left[k\right]}^{r}\right|}$$

(24) $$L_{V2R}=-\log\frac{\;exp\left(q_{i}^{v}\cdot \varphi_{\hat{y_{i}^{v}}}^{\mathrm{rs}}/\tau \right)}{\sum_{k=0}^{N^{r}}exp\left(q_{i}^{v}\cdot \varphi_{k}^{\mathrm{rs}}/\tau \right)}$$

(25) $$\varphi_{k}^{\mathrm{rs}}=\frac{\left(\sum_{u_{n}^{r}\in H_{k}^{r}}u_{n}^{r}+\sum_{u_{n}^{v}\in H_{R2V\left[k\right]}^{v}}u_{n}^{v}\right)}{\left|H_{k}^{r}\cup H_{R2V\left[k\right]}^{v}\right|}$$

(26) $$L_{R2V}=-\log\frac{\;exp(q_{i}^{r}\cdot \varphi_{\hat{y_{i}^{r}}}^{\mathrm{vs}}/\tau )}{\sum_{k=0}^{N^{v}}exp\left(q_{i}^{r}\cdot \varphi_{k}^{\mathrm{vs}}/\tau \right)}$$

In this context, $\varphi_{k}^{\mathrm{vs}}$ and $\varphi_{k}^{\mathrm{rs}}$ denote the modality-specific shared memories for the visible-light and infrared modalities, respectively. Additionally, $\hat{y_{i}^{v}}=V2R[y_{i}^{v}]$ and $\hat{y_{i}^{r}}=R2V[y_{i}^{r}]$ refer to the cross-modality labels for the visible-light query sample $q_{i}^{v}$ and the infrared query sample $q_{i}^{v}$, respectively. Furthermore, $H_{V2R\left[k\right]}^{r}$ and $H_{R2V\left[k\right]}^{v}$ represent the set of infrared cluster instances corresponding to the k-th visible-light cluster, or the set of visible-light cluster instances corresponding to the k-th infrared cluster, respectively. $H_{k}^{v}$ represents the set of instances contained in the k-th cluster in the visible light modality. $H_{k}^{r}$ represents the set of instances contained in the k-th cluster in the infrared light modality. τ denotes the temperature coefficient.

The total loss function for modal shared contrastive learning is expressed as $L_{\mathrm{scl}}=L_{V2R}+L_{R2V}$. This function aids $L_{\mathrm{id}}$ in the reconstruction process.

### 3.4. Varied Regional Neighbor Learning

After completing cross-modal regional alignment and feature distribution optimization in the VRA module, we introduce the VRNL module to further improve neighbor-learning reliability and cross-modal feature consistency. VRNL combines multi-region spatial association with parameter constraints to perform efficient neighbor mining. For the query samples $q_{i}^{v}$ and $q_{i}^{r}$, we define the intra-modal neighbor set as follows:

(27) $$N^{v}\left(q_{i}^{v}\right)=\left\{N^{v}{\left(q_{i}^{v}\right)}^{\left(1\right)}\cap \cdots \cdots \cap {\;N}^{v}{\left(q_{i}^{v}\right)}^{\left(N\right)}\right\}$$

(28) $$N^{r}\left(q_{i}^{r}\right)=\left\{N^{r}{\left(q_{i}^{r}\right)}^{\left(1\right)}\cap \cdots \cdots \cap \;N^{r}{\left(q_{i}^{r}\right)}^{\left(N\right)}\right\}$$

In this context, $N^{m}{\left(q_{i}^{m}\right)}^{\left(k\right)}$ represents the intra-modal neighbor set derived from the k-th domain space of $q_{i}^{m}$. The variable m can be either v or r. The definition of $N^{m}{\left(q_{i}^{m}\right)}^{\left(k\right)}$ is as follows:

(29) $$N^{m}{\left(q_{i}^{m}\right)}^{\left(k\right)}=\left\{u_{j}^{m\left(k\right)}\mathrm{sim}\left(q_{i}^{m(k)},u_{j}^{m\left(k\right)}\right)\geq \rho \cdot \underset{j=1\ldots N_{m}}{\max}\mathrm{sim}\left(q_{i}^{m(k)},u_{j}^{m\left(k\right)}\right)\right\},m\in \left\{v,r\right\}$$

Among them, *ρ* represents the size of the range that neighbors can choose from.

For the query samples $q_{i}^{v}$ and $q_{i}^{r}$, the inter-modal neighbor sets are defined as follows:

(30) $$N^{r}\left(q_{i}^{v}\right)=\left\{N^{r}{\left(q_{i}^{v}\right)}^{\left(1\right)}\cap \cdots \cdots \cap N^{r}{\left(q_{i}^{v}\right)}^{\left(N\right)}\right\}$$

(31) $$N^{v}\left(q_{i}^{r}\right)=\left\{N^{v}{\left(q_{i}^{r}\right)}^{\left(1\right)}\cap \cdots \cdots \cap N^{v}{\left(q_{i}^{r}\right)}^{\left(N\right)}\right\}$$

For $N^{r}{\left(q_{i}^{v}\right)}^{\left(k\right)}$, the inter-modal neighbor set is derived from the k-th domain space of $q_{i}^{v}$. The same principle applies to $N^{v}\left(q_{i}^{r}\right)$. The definitions of $N^{r}{\left(q_{i}^{v}\right)}^{\left(k\right)}$ and $N^{v}{\left(q_{i}^{r}\right)}^{\left(k\right)}$ are as follows:

(32) $$N^{r}{\left(q_{i}^{v}\right)}^{\left(k\right)}=\left\{u_{j}^{r\left(k\right)}\mathrm{sim}\left(q_{i}^{v(k)},u_{j}^{r\left(k\right)}\right)>\rho \cdot \underset{j=1\ldots N^{r}}{\max}\mathrm{sim}\left(q_{i}^{v(k)},u_{j}^{r\left(k\right)}\right)\right\}$$

(33) $$N^{r}{\left(q_{i}^{v}\right)}^{\left(k\right)}=\left\{u_{j}^{r\left(k\right)}\mathrm{sim}\left(q_{i}^{v(k)},u_{j}^{r\left(k\right)}\right)>\rho \cdot \underset{j=1\ldots N^{r}}{\max}\mathrm{sim}\left(q_{i}^{v(k)},u_{j}^{r\left(k\right)}\right)\right\}$$

In this way, we obtain the neighbor sets $N^{v}\left(q_{i}^{v}\right)$, $N^{r}\left(q_{i}^{r}\right)$, $N^{r}\left(q_{i}^{v}\right)$, $N^{v}\left(q_{i}^{r}\right)$.

We sequentially optimize the obtained neighbor sets $N^{v}\left(q_{i}^{v}\right)$, $N^{r}\left(q_{i}^{r}\right)$, $N^{r}\left(q_{i}^{v}\right)$, $N^{v}\left(q_{i}^{r}\right)$. Inspired by CALR [41], we developed an optimization method specifically for these neighbor sets. For a given query sample $q_{i}^{v}$, we derive the neighbor set $N^{v}\left(q_{i}^{v}\right)$, the corresponding local cluster $L\left(q_{i}^{v}\right)$ formed within the camera, and multiple sub-clusters $N^{v}{\left(q_{i}^{v}\right)}_{c=0}^{\mathrm{num}\_c}$ segmented from $N^{v}\left(q_{i}^{v}\right)$. Thus, the optimization process for the neighbor set $N^{v}\left(q_{i}^{v}\right)$ is expressed as follows:

(34) $${\;N}^{v}\left(q_{i}^{v}\right)=N^{v}{\left(q_{i}^{v}\right)}_{c=g}\cap L\left(q_{i}^{v}\right)\cup \left(N^{v}\left(q_{i}^{v}\right)\backslash N^{v}{\left(q_{i}^{v}\right)}_{c=g}\right),i=0,\ldots ,\mathrm{num\_c}$$

In this study, $N^{v}\left(q_{i}^{v}\right)\backslash N^{v}{\left(q_{i}^{v}\right)}_{c=g}$ denotes the sample set remaining after excluding samples captured by camera c from the neighbor set $N^{v}\left(q_{i}^{v}\right)$. This method effectively removes noisy samples from the same camera, thereby enhancing the reliability of the neighbor set results. The same approach is applied to optimize $N^{r}\left(q_{i}^{r}\right)$, $N^{r}\left(q_{i}^{v}\right)$, and $N^{v}\left(q_{i}^{r}\right)$.

For the given query sample $q_{i}^{v}$, the neighbor learning loss from visible light to visible light is determined using the following formula:

(35) $$S_{\mathrm{pos}}\left(i,j\right)=\exp(\frac{\mathrm{sim}\left(q_{i}^{v},u_{j}^{v}\right)}{\tau })$$

(36) $$S_{\mathrm{total}}\left(i\right)=\overset{N^{v}}{\underset{k=1}{\sum }}\exp(\frac{\mathrm{sim}\left(q_{i}^{v},u_{k}^{v}\right)}{\tau })$$

(37) $$L_{\sin\mathrm{gle}}\left(i,j\right)=-\log\left(\frac{S_{\mathrm{pos}}\left(i,j\right)}{S_{\mathrm{total}}\left(i\right)}\right)$$

(38) $$L_{\mathrm{vv}}=\frac{\sum_{i=1}^{N^{\mathrm{batch}}}\sum_{j\in N^{v}\left(q_{i}^{v}\right)}L_{\sin\mathrm{gle}}\left(i,j\right)}{N^{\mathrm{batch}}\left|N^{v}\left(q_{i}^{v}\right)\right|}$$

In this context, $N^{batch}$ denotes the batch size of the query sample $q_{i}$. $S_{pos}\left(i,j\right)$ signifies the similarity contribution of the positive samples and $S_{total}\left(i\right)$ indicates the cumulative similarity contributions between the query samples and all visible-light samples. Additionally, $L_{single}\left(i,j\right)$ indicates the loss of a single query–neighbor pair. $L_{vv}$ implies the total neighbor learning loss from visible light to visible light. Similarly, we calculate the neighbor learning losses for transitions from visible light to infrared, infrared to infrared, and infrared to visible light, represented as $L_{vr}$, $L_{rr}$, and $L_{rv}$, respectively. Ultimately, the final optimization objective of Varied Regional Neighbor Learning is defined by the following formula:

(39) $$L_{NL}=L_{vv}+L_{vr}+L_{rr}+L_{rv}$$

### 3.5. Uniform Merging

Following the above processing, there is a significant similarity among multiple visible light clusters linked to the same infrared cluster, and vice versa. These clusters likely originate from the same ID split. Thus, we apply Uniform Merging to combine different clusters of the same identity in both visible light and infrared modalities, further minimizing intra-modal differences.

To retrieve infrared clusters corresponding to a visible-light query feature $q^{v}$, first identify the infrared clusters linked to $q^{v}$. Next, find all visible-light clusters associated with these infrared clusters. This process yields a set of visible-light clusters that share the same pedestrian identity as the visible-light query feature $q^{v}$. We denote the number of clusters in this group as $N_{cluster}^{v}$. Thus, multiple distinct visible-light clusters are represented by the same cross-modal label, $q^{v}\left(i\right)=R2V[V2R[y^{v}]]\left[i\right](i<N_{cluster}^{v})$.

The alternating contrastive learning strategy [18] facilitates the Uniform Merging process using the visible-light modality-specific memory $\varphi_{k}^{v}$.

(40) $$L_{i\to j}^{v}=-\log\frac{\exp\left(q^{v}\left(i\right)\cdot \phi_{y^{v}\left(j\right)}^{v}/\tau \right)}{\sum_{k=0}^{N_{\mathrm{cluster}}^{v}}\exp\left(q^{v}\left(i\right)\cdot \phi_{k}^{v}/\tau \right)}$$

(41) $$L_{\mathrm{mate}}^{v}=\left\{\begin{array}{ll} \overset{N_{\mathrm{cluster}}^{v}-2}{\underset{i=0}{\sum }}L_{i\to \left(i+1\right)}^{v}, & \mathrm{if}\;\mathrm{Epoch}\%2=0\\ \overset{N_{\mathrm{cluster}}^{v}-2}{\underset{i=0}{\sum }}L_{\left(i+1\right)\to i}^{v}, & \mathrm{if}\;\mathrm{Epoch}\%2=1 \end{array}\right.\;$$

For the infrared modality, consider an infrared query feature denoted as $q^{r}$. We assume the number of clusters in the infrared group, associated with the same pedestrian identity as $q^{r}$, equals $N_{cluster}^{r}$. These clusters are represented with the same cross-modal label as $q^{r}\left(p\right)=V2R[R2V[y^{r}]]\left[p\right](p<N_{cluster}^{r})$.

Similarly, we can obtain

(42) $$L_{p\to q}^{r}=-\log\frac{\exp\left(q^{r}\left(p\right)\cdot \phi_{y^{r}\left(q\right)}^{r}/\tau \right)}{\sum_{k=0}^{N_{\mathrm{cluster}}^{r}}\exp\left(q^{r}\left(p\right)\cdot \phi_{k}^{r}/\tau \right)}$$

(43) $$L_{\mathrm{mate}}^{r}=\left\{\begin{array}{ll} \overset{N_{\mathrm{cluster}}^{r}-2}{\underset{p=0}{\sum }}L_{p\to \left(p+1\right)}^{r}, & \mathrm{if}\;\mathrm{Epoch}\%2=0\\ \overset{N_{\mathrm{cluster}}^{r}-2}{\underset{p=0}{\sum }}L_{\left(p+1\right)\to p}^{r}, & \mathrm{if}\;\mathrm{Epoch}\%2=1 \end{array}\right.\;$$

To balance the contributions of the two modality fusion losses, we introduce hyperparameters $\delta_{1}$ and $\delta_{2}$. The total loss for Uniform Merging is defined as

(44) $$L_{mate}=\delta_{1}\cdot L_{mate}^{v}+\delta_{2}\cdot L_{mate}^{r}$$

Among these, $\delta_{1}>0$ and $\delta_{2}>0$ serve as adjustable parameters to control the proportions of visible light fusion loss $L_{mate}^{v}$ and infrared fusion loss $L_{mate}^{r}$ within the total loss.

The final total training loss can be expressed as

(45) $$L=L_{\mathrm{id}}+L_{mc}+\alpha_{1}L_{scl}+\alpha_{2}L_{NL}+\alpha_{3}L_{mate}$$

## 4. Experiments

### 4.1. Datasets

This study evaluated the proposed method’s performance using two well-known benchmark datasets in visible–infrared person re-identification (VI-ReID): SYSU-MM01 [42] and RegDB [43]. The experiments adhered to established protocols in the field.

SYSU-MM01 is a comprehensive cross-modal pedestrian dataset, gathered from various indoor and outdoor environments using a multi-camera system. This system includes four visible light (RGB) cameras and two near-infrared (IR) cameras. The dataset comprises 303,420 pedestrian images representing 491 unique identities, with 287,628 images in the visible light modality and 15,792 in the infrared modality. Significant intra-class variations exist in the images for each identity, due to real-world factors such as changes in view angles, pedestrian poses, lighting conditions, and occlusions. These variations effectively simulate the challenges encountered in the VI-ReID task.

SYSU-MM01 is structured into distinct training and test sets for model training and evaluation. The training set comprises 395 identities, including 22,258 visible light images and 11,909 infrared images in certain protocol settings. The test set consists of 96 identities. During inference, the query set includes 3803 infrared images from the test set, while the gallery set contains 301 randomly selected visible light images. Two standard evaluation modes are used: (1) All-Search mode, where the gallery set is randomly sampled from visible light images taken by both indoor and outdoor cameras; (2) Indoor-Search mode, where the gallery set consists solely of visible light images captured by indoor cameras.

RegDB is a compact yet representative cross-modal pedestrian dataset captured using a dual-camera system comprising a visible light (RGB) camera and an infrared (IR) camera. The dataset includes 8240 images representing 412 unique identities, with each identity having 10 visible light images and 10 infrared images, totaling 4120 images for each modality. Like SYSU-MM01, RegDB exhibits significant intra-class variations due to real-world factors such as pose, illumination, and viewing angle.

RegDB was randomly split into two non-overlapping subsets for training and testing, each with 206 identities. Following established studies, we employed two standard cross-modal evaluation modes: (1) Visible-to-Infrared/Visible–Thermal mode, which involves retrieving matching infrared images using visible-light images as queries, and (2) Infrared-to-Visible/Thermal–Visible mode, which involves retrieving matching visible-light images using infrared images as queries.

We employed standard experimental settings to assess the proposed method on the two benchmark datasets [44], and three core performance metrics, Cumulative Matching Characteristics (CMCs, quantified by Rank-1 accuracy), mean Average Precision (mAP), and mean Inverse Negative Penalty (mINP [2]), were adopted for comprehensive evaluation. These metrics are the de facto standards in the VI-ReID field, ensuring a fair and rigorous comparison with existing state-of-the-art methods.

To quantitatively evaluate the cross-modal retrieval performance of the proposed method, three widely adopted and standard metrics in the person re-identification community are employed: Rank-1 accuracy, mean Average Precision (mAP) and mean Inverse Negative Penalty (mINP). Their specific definitions in unsupervised visible–infrared person re-identification (USL-VI-ReID) are elaborated as follows.

Rank-1 accuracy: It is the most intuitive retrieval metric, representing the percentage of queries for which the top-1 retrieved gallery sample is the correct match (the same identity as the query).

mean Average Precision (mAP): It is the mean value of Average Precision (AP) across all query samples, where AP measures the precision of all correct retrievals for a single query at all recall levels. Mathematically, AP is calculated as the area under the precision–recall curve for each query, and mAP further averages this metric over the entire query set.

mean Inverse Negative Penalty (mINP): It is a challenging metric designed to evaluate the model’s retrieval performance on hard samples (e.g., pedestrians with severe occlusions, large pose changes or low image quality). mINP is defined as the mean value of the inverse of the negative penalty for each query, where the negative penalty is determined by the ratio of the number of incorrect samples ranked higher than the first correct sample to the total number of gallery samples.

To further evaluate the robustness of the proposed method under different similarity thresholds, we also supplement the Receiver Operating Characteristic (ROC) curve, which plots the True Positive Rate (TPR) against the False Positive Rate (FPR) to reflect the trade-off between retrieval accuracy and false positive risk.

### 4.2. Implementation Details

This method utilizes the PyTorch framework and was trained on four NVIDIA GeForce RTX 4090 graphics cards (GPU Part Number: 2684-301-A1; max boost clock: 3105 MHz; memory: 24,564 MiB GDDR6X). We employed the Visual Transformer (Vit) [45] as the backbone network. Unlike CNNs, which expand local receptive fields, the ViT self-attention mechanism directly models long-range dependencies across the entire image [45]. This capability is essential for capturing both global pedestrian structure (for example, body size) and local discriminative features (for example, clothing texture), thereby addressing the core problem of insufficient feature granularity in USL-VI-ReID. 

During the training phase, we selected four identities, each with 16 instances. We applied preprocessing techniques including random horizontal flipping, cropping, occlusion, color jittering, and channel erasing. All pedestrian images for training and testing were resized to the specified size of 384 × 128.

The initial learning rate is set to $3.5\times 10^{-4}$. It decreases by a factor of 0.1 every 20 epochs. The training process adopts a two-stage progressive optimization strategy to balance feature extraction, modal alignment, and cluster refinement. We adopt the Stochastic Gradient Descent (SGD) optimizer for model training, which is widely used in person re-identification tasks for stable convergence. The training process uses different learning rates and fixed weight decay for the two phases. The detailed design logic and implementation steps are as follows:

Phase 1 (Epoch 1–50): The initial learning rate is set to $3.5\times 10^{-4}$, with momentum = 0.9 and weight decay = $5\times 10^{-4}$. This phase focuses on feature initialization and modal gap mitigation. Only PA-CBAM and MAAL modules are enabled. PA-CBAM is used to extract fine-grained identity features, while MAAL performs three-modal adversarial learning to align the feature distributions of visible, infrared, and CA modalities. This phase lays a foundation for subsequent cross-modal association by reducing inherent modal discrepancies.

Phase 2 (Epoch 51–100): The learning rate is reduced to $3.5\times 10^{-5}$, while momentum and weight decay remain unchanged. The momentum parameter accelerates the convergence of SGD in flat regions of the loss function, and the fixed weight decay suppresses over-fitting caused by pseudo-label noise in unsupervised learning, which is critical for the stability of the multimodule collaborative framework. This phase focuses on cross-modal alignment and cluster optimization. On the basis of Phase 1, VRA, VRNL, and UM modules are added. VRA realizes regional-level cross-modal feature matching, VRNL enhances the reliability of neighbor learning and stabilizes pseudo-labels, and UM merges split clusters of the same identity. This progressive module activation strategy avoids training instability caused by excessively complex constraints at the initial stage.

We set the batch size to 64, organized as four identities per batch with 16 instances for each identity. Input images have a height of 384 and a width of 128. For data augmentation, we apply random horizontal flipping with probability 0.5, random cropping to a target size of 384 × 128, color jittering (brightness = 0.5, contrast = 0.5, saturation = 0.5), and channel erasing with probability 0.5. The hyperparameters α and β are set to 1.0 and 0.1, respectively. Similarly, δ_1_ and δ_2_ are assigned values of 0.6 and 0.4. For α_1_, α_2_, and α_3_, the values are 0.4, 0.5, and 0.03. According to [18,34], for intra-modal clustering on two datasets, the hyperparameters $k_{1}$ and $k_{2}$ are both set to 30 and 6. In inter-modal clustering, to thoroughly explore inter-modal associations, $k_{1}$ and $k_{2}$ are set to 40 and 32 on the SYSU-MM01 dataset, while on the RegDB dataset, they are set to 38 and 18. The parameter μ, which limits the number of matches, is set to 3. This prevents excessive inter-modal matching while maximizing its potential. The number of regions N is dataset-specific: four for SYSU-MM01 and six for RegDB. The neighborhood radius for DBSCAN is consistently set to 0.6 across both datasets.

### 4.3. Comparison with State-of-the-Art Methods

In this subsection, we compare our proposed method with the current state-of-the-art techniques. These include 11 supervised visible–infrared person re-identification (SVI-ReID) methods, 6sixunsupervised single-modal person re-identification (USL-ReID) methods, and 15 unsupervised visible–infrared person re-identification (USL-VI-ReID) methods. Table 1 presents the comparison results.

#### 4.3.1. Performance Comparison on the SYSU-MM01 Dataset

Our method significantly surpasses existing unsupervised single-modal person re-identification (USL-ReID) techniques. USL-ReID methods, while excelling in single-modal tasks [3,20,33,58], fail to account for inter-modal differences, resulting in poor performance in cross-modal person re-identification. Consequently, USL-ReID is unsuitable for cross-modal applications.

Our method surpasses current state-of-the-art unsupervised methods. On the SYSU-MM01 dataset’s “all search” mode, it achieves a Rank-1 accuracy of 66.20%, a mean Average Precision (mAP) of 62.79%, and a mean Inverse Negative Penalty (mINP) of 49.42%. In “Indoor Search” mode, these metrics improve to 69.36%, 74.72%, and 70.84%, respectively. Compared to the leading unsupervised method, DMDL [68], our approach shows superior performance on the RegDB dataset.

Our method surpasses some supervised cross-modal person re-identification (SVI-ReID) techniques. This improvement stems from effectively leveraging information across different region labels. However, it still lags behind the current state-of-the-art fully supervised methods. This performance gap is primarily due to the absence of annotation information in the unsupervised approach.

#### 4.3.2. Performance Comparison on the RegDB Dataset

Our method not only surpasses unsupervised single-modal person re-identification (USL-ReID) techniques but also achieves state-of-the-art performance in unsupervised cross-modal person re-identification (USL-VI-ReID). Specifically, for the visible-to-infrared and infrared-to-visible conversion modes, it recorded 93.34% and 91.89% on Rank-1, 87.55% and 86.58% on mAP, and 76.08% and 73.60% on mINP, respectively. When compared to the current leading unsupervised method, DMDL [70], on the RegDB dataset, our approach shows superior performance. Notably, in the visible-to-infrared mode, it improves by 2.71% on Rank-1, 2.22% on mAP, and 2.29% on mINP.

Furthermore, our method outperforms certain supervised cross-modal person re-identification (SVI-ReID) methods on the RegDB dataset. This result further confirms the effectiveness of our proposed approach.

We evaluate the computing time on a NVIDIA GeForce RTX 4090. Our input size measures 384 × 128, and our method contains approximately 91.5 million parameters, which is about 5 million more than the standard ViT-Base model. The model achieves 31.6 GFLOPs on FLOPs parameters. Table 2 indicates that there is still potential for improving FPS, and we will explore further lightweight options for the model in the future.

Compared with clustering-based USL-VI-ReID methods such as RULN [66], our approach incurs a higher computational cost. Clustering-only methods typically optimize a simple contrastive loss and omit complex adversarial training or multi-region feature alignment; therefore, their overhead remains low, comparable to the ResNet-50 baseline reported in Table 2. This lower cost, however, comes at the expense of cross-modal alignment: clustering-only pipelines frequently fail to bridge severe modality gaps, whereas the extra computation of our method delivers substantially better cross-modal alignment and downstream discriminability.

Relative to adversarial-based USL-VI-ReID approaches, our hybrid framework offers clear efficiency advantages. Conventional GAN-based solutions commonly train multiple generators and discriminators, which markedly increases computational demand; by contrast, our lightweight MAAL module achieves similar alignment objectives with a much lower cost. Although multimodule collaboration reduces our FPS compared with the basic ViT-Base, it does not produce the exponential FLOPs growth characteristic of heavy adversarial models. Thus, our method attains a pragmatic trade-off, preserving much of the discriminative power associated with adversarial learning while maintaining efficiency closer to clustering-based approaches.

**Figure 5:**
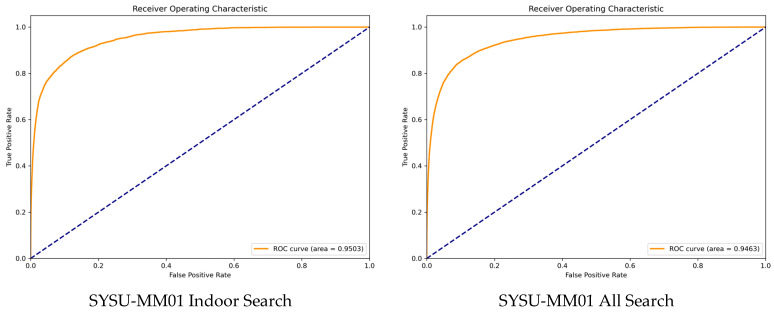
ROC curves of the proposed method on SYSU-MM01 datasets. The dashed line denotes the random guess baseline. AUC values are labeled in the legend to quantify the retrieval robustness.

Figure 5 presents the ROC curves of our method on SYSU-MM01 datasets, with the dashed line representing the random guess baseline. It can be observed that our method’s curves are significantly above the baseline across all scenarios, indicating strong discriminative ability to distinguish positive cross-modal pairs from negative ones. Specifically, the Area Under the Curve (AUC) values reach 95.03% (SYSU-MM01 Indoor Search) and 94.63% (SYSU-MM01 All Search), which verifies the superior robustness of our method under different similarity thresholds. This performance gain benefits from the multimodule collaborative optimization, which enhances the model’s ability to capture consistent cross-modal cues while suppressing false matches.

### 4.4. Ablation Study

In this subsection, we verify the effectiveness of each component of the proposed method through rigorous ablation experiments. Table 3 presents the experimental results.

#### 4.4.1. Validation of the Effectiveness of PA-CBAM

Comparing the results of No. 2 and No. 1 reveals that introducing PA-CBAM to the baseline model improved its Rank-1 accuracy by over 1 percentage point. PA-CBAM’s primary function is to enhance pedestrian feature discriminability and accuracy. It achieves this through a two-level attention mechanism involving “channel dimension optimization and spatial dimension adjustment”. This mechanism provides a high-quality feature foundation for subsequent modules like cross-modality alignment and neighbor learning.

#### 4.4.2. Validation of the Effectiveness of VRA

Comparing the results of No. 5 and No. 1 reveals that introducing the VRA improved model performance by over 10% on the SYSU-MM01 dataset and over 37% on the RegDB dataset. This improvement primarily stems from the MAAL module’s role as an auxiliary component. The MAAL module reduces interference from inherent modal differences by enhancing crucial identity-discriminating feature channels and suppressing redundant noise channels. Consequently, the VRA module more effectively captures “essentially consistent regional features” across modalities. It also identifies and matches diverse regional features between different modalities, ultimately reducing modal differences and laying the groundwork for subsequent cross-modal association tasks.

#### 4.4.3. Validation of the Effectiveness of VRNL

Comparing No. 4 with No. 1 reveals that using VRNL alone significantly enhances performance. Furthermore, as indicated by No. 6, incorporating VRNL with VRA further boosts performance. The model’s ability to learn from neighbors improves by leveraging multi-region spatial correlation. Additionally, the integration of the PA-CBAM module within the VRNL module ensures that multi-region features are not affected by low-quality features, thereby enhancing the accuracy of neighbor learning.

#### 4.4.4. Validation of the Effectiveness of UM

Serial numbers 3 and 7 demonstrate that incorporating UM into the baseline model, either alone or alongside VRA and VRNL, enhances model performance. This improvement occurs because UM consolidates scattered clustering of identical IDs, reduces noise, and offers a unified ID feature representation. Consequently, it addresses the fragmentation of identical ID features left by previous modules, thereby boosting the model’s performance, quality, and robustness.

To further clarify the core functions and unique contributions of each module and the CA modality in the proposed framework, their respective design objectives, solved challenges and key contributions are summarized in Table 4.

### 4.5. Hyperparameter Analysis

**Figure 6:**
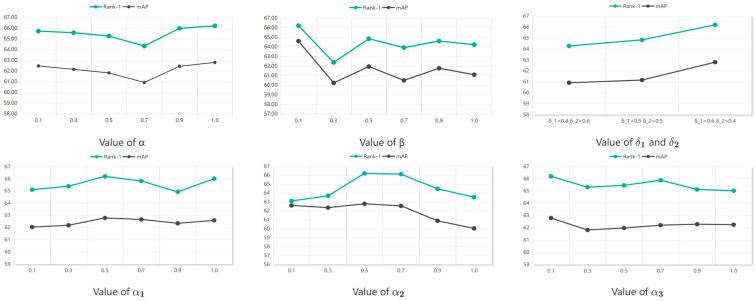
Performance of our framework with different values of α, β, $\delta_{1}$ and $\delta_{2}$, $\alpha_{1}$, $\alpha_{2}$, $\alpha_{3}$ on SYSU-MM01 datasets.

The hyperparameters α and β in formula (17) regulate the adversarial intensity between the “backbone network” and the “modality classifier.” In the UM module, the hyperparameters $\delta_{1}$ and $\delta_{2}$ in formula (44) balance the fusion loss of the two modalities. Additionally, the hyperparameters α_1_, α_2_, and α_3_ in formula (45) adjust the weight parameters of VRA, VRNL, and UM. As illustrated in Figure 6, we evaluated the model’s performance across various parameter values.

In addition to the hyperparameters analyzed above, the regularization weight λ in the PA-CBAM module is another key hyperparameter affecting model performance, and its optimal value is determined through grid search experiments combined with performance convergence verification, the representative dataset for USL-VI-ReID. Specifically, for λ, we search the grid range of [0.001, 0.01, 0.1, 1.0, 10.0] and select the value with the highest Rank-1 accuracy of the cross-modal retrieval; the optimal value of λ = 0.1 is finally determined. Initially, the value of α was set to 1.0. We then adjusted β to 0.1, 0.3, 0.5, 0.9, and 1.0. The results indicated that decreasing β improved model performance. However, when β reached 0.1, the performance gains plateaued, making the model performance relatively insensitive to further changes in α. Consequently, α was set to 1.0.

Regarding $\delta_{1}$ and $\delta_{2}$, previous research has sometimes assigned equal contribution weights of 0.5 to the visible and infrared modalities; this practice has been recommended because balancing the weights can improve the extraction of infrared features when the visible-light dataset is substantially larger than the infrared dataset. In contrast, our experiments showed that weighting the visible and infrared modalities at 0.6 and 0.4, respectively, produced superior performance compared with the 0.5/0.5 split. We attribute this improvement to the greater amount of fine-grained feature information present in the visible-light modality relative to the infrared modality.

For the weighting parameters $\alpha_{1}$, $\alpha_{2}$, and $\alpha_{3}$, the model achieved optimal performance when $\alpha_{1}$ equaled 0.5. Experimental observations indicated that the model was relatively insensitive to the values of $\alpha_{2}$ and $\alpha_{3}$: setting $\alpha_{2}$ = 0.5 and $\alpha_{3}$ = 0.1 produced the best performance.

### 4.6. Analysis of Visualization

**Figure 7:**
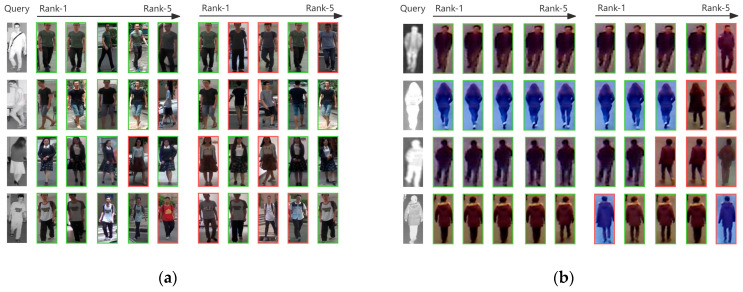
(**a**) Top 5 retrieval results on SYSU-MM01 datasets. Our model demonstrates superior cross-modal retrieval capability compared to the baseline; (**b**) Top 5 retrieval results on RegDB datasets. Our model demonstrates superior cross-modal retrieval capability compared to the baseline.

Visualization of retrieval results: Figure 7 compares the retrieval outcomes of our method with the baseline method. The baseline model demonstrates limited retrieval capabilities. In contrast, our method accurately retrieves data even in complex scenarios involving variations in color, texture, perspective, and environment.

**Figure 8:**
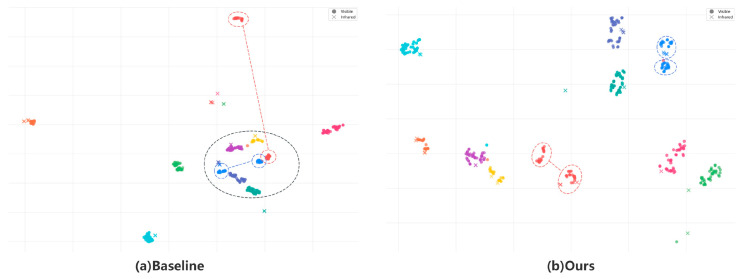
The t-SNE visualization displays 10 randomly selected identities. Colors represent different identities, with circles indicating the visible modality and crosses indicating the infrared modality.

T-SNE Feature Visualization: Figure 8 illustrates the t-SNE feature distribution of 10 randomly selected identity samples from the SYSU-MM01 dataset. In contrast to the baseline model, samples of the same identity are more closely clustered in the feature space, while samples of different identities show clearer and more distinct separation. This finding confirms our method’s effectiveness in cross-modal feature alignment and improving the identity discriminability of features.

## 5. Conclusions

This paper presents a multimodule collaborative optimization framework that addresses three principal challenges in unsupervised visible–infrared person re-identification (USL-VI-ReID): large modal discrepancies, limited feature granularity, and underutilization of channel augmentation (CA). Treating CA as an independent input modality, the framework achieves improvements through four complementary modules that interact synergistically. First, the PA-CBAM module implements a two-level attention strategy that initially locates pedestrian regions and then refines their representations, thereby extracting precise local identity cues. Second, the VRA module, together with the multimodal auxiliary adversarial learning (MAAL) module, mitigates modal gaps and promotes cross-spectral feature alignment. Third, the VRNL module based on the cross-modal aligned features enforces reliable neighborhood learning by exploiting multi-region spatial associations. Finally, the UM module increases robustness to interference by merging split clusters corresponding to the same identity. Extensive experiments on the SYSU-MM01 and RegDB datasets validate the approach: it substantially outperforms most unsupervised baselines and even exceeds the performance of several supervised methods.

Despite promising results, the method retains several important limitations that warrant further improvement. First, the framework degrades under extreme environmental conditions—for example, heavy rain, dense fog, or severe occlusion from objects such as umbrellas and backpacks. The PA-CBAM module’s multi-scale spatial attention depends on relatively clear regional features; when critical body parts (for instance, the head and torso) are heavily occluded, the VRA module struggles to align cross-modal information and to extract consistent local cues. Second, the model exhibits higher computational complexity than lightweight USL-VI-ReID approaches. Integrating four collaborative modules with a ViT backbone increases parameter count and floating-point operations (FLOPs), which constrains deployment on resource-limited edge devices (such as low-power surveillance cameras and drones) that require strict hardware budgets for real-time inference. Third, the framework’s hyperparameters are dataset-specific (for example, the number of regions N equals 4 for SYSU-MM01 and 6 for RegDB) and lack an adaptive tuning mechanism for unseen datasets. This dependence weakens generalization in practical settings, where dataset characteristics—image resolution, number of identities, and intra-class variation—may differ substantially.

Based on the identified limitations, we propose three promising directions for future work. First, we improve discriminative feature extraction under extreme conditions by integrating a deformable attention mechanism into the PA-CBAM module. This integration allows the model to adapt its receptive field dynamically to occlusion patterns and environmental noise and, when combined with part-aware self-supervised pretraining tasks (for example, pedestrian part segmentation), strengthens the robustness of local feature representations. Second, we investigate model-lightweighting strategies to increase deployment feasibility. Concretely, we replace the full ViT backbone with a hybrid architecture (for example, a CNN–ViT fusion) that preserves CNN strengths in local feature extraction while exploiting Transformer capabilities for global dependency modeling. We also adopt knowledge distillation to compress the multimodule framework, transferring discriminative knowledge from the current large model to a lightweight student network with minimal performance loss. Third, we develop an adaptive hyperparameter-optimization framework driven by dataset meta-features such as average image resolution, occlusion rate, and modal-difference intensity. A trained meta-regressor predicts optimal hyperparameters (for example, the number of regions N and the DBSCAN neighborhood radius) for new datasets, thereby improving the model’s generalization across diverse real-world scenarios.

Future research efforts not only address the current method’s limitations but also advance the practical deployment of USL-VI-ReID in more complex, resource-constrained intelligent surveillance scenarios.

## Figures and Tables

**Table 1:** Comparison with SOTA on SYSU-MM01 and RegDB. Rank at r accuracy (%), mAP (%) and mINP (%) are reported. The red, blue and green, respectively, represent the 1^st^, 2^nd^ and 3^rd^ in the USL-VI-REID task.

	Methods	Venue	SYSU-MM01						RegDB					
			All Search			Indoor Search			Visible to Infrared			Infrared to Visible		
			Rank-1	mAP	mINP	Rank-1	mAP	mINP	Rank-1	mAP	mINP	Rank-1	mAP	mINP
SVI-ReID	CA [46]	ICCV-21	69.88	66.89	53.61	76.26	80.37	76.79	85.03	79.14	65.33	84.75	77.82	61.56
	MSO [47]	MM-21	58.70	56.42	-	63.09	70.31	-	73.6	66.9	-	74.6	67.5	-
	MCLNet [22]	ICCV-21	65.40	61.98	47.39	72.56	76.58	72.10	80.31	73.07	57.39	75.93	69.49	52.63
	NFS [48]	CVPR-21	56.91	55.45	-	62.79	69.79	-	80.5	72.1	-	78.0	69.8	-
	FMCNet [24]	CVPR-22	66.34	62.51	-	68.15	74.09	-	89.12	84.43	-	88.38	83.86	-
	MAUM [23]	CVPR-22	71.68	68.79	-	76.97	81.94	-	87.87	85.09	-	86.95	84.34	-
	DMiR [49]	TCSVT-22	50.54	49.29	-	53.92	62.49	-	75.79	69.97	-	73.93	68.22	-
	DART [50]	CVPR-22	68.72	66.29	53.26	72.52	78.17	74.94	83.6	75.7	60.60	81.97	73.78	56.70
	TMD [51]	TMM-23	68.81	63.96	48.11	76.31	74.52	65.05	87.04	81.19	68.73	83.54	77.92	64.33
	PartMix [52]	CVPR-23	77.8	74.6	-	81.5	84.4	-	85.7	82.3	-	84.9	82.5	-
	DEEN [53]	CVPR-23	74.7	71.8	-	80.3	83.3	-	91.1	85.1	-	89.5	83.4	-
	MFRNet [54]	CVPR-24	79.2	76.5	58.3	83.1	85.7	78.2	92.5	88.3	75.1	91.8	87.6	73.5
	IDKL [55]	CVPR-24	81.42	79.85	-	87.14	89.37	-	94.72	90.19	-	94.22	90.43	-
	DCMAN [56]	TIFS-25	76.4	73.8	56.1	80.2	83.9	76.4	90.8	86.5	72.3	89.9	85.8	70.6
	TSKD [57]	PR-25	76.6	73.0	-	82.7	85.3	-	91.1	81.7	-	89.9	80.5	-
USL-ReID	CAP [3]	AAAI-21	16.82	15.71	7.02	24.57	30.74	26.15	9.71	11.56	8.74	10.21	11.34	7.92
	IICS [32]	CVPR-21	14.39	15.74	8.41	15.91	24.87	22.15	14.39	15.74	8.41	15.91	24.87	22.15
	ICE [31]	ICCV-21	20.54	20.39	10.24	29.81	38.35	34.32	12.98	15.64	11.91	12.18	14.82	10.60
	Cluster-Contrast [20]	ACCV-22	20.16	22.00	12.97	23.33	34.01	30.88	11.76	13.88	9.94	11.14	12.99	8.99
	PPLR [33]	CVPR-22	12.58	12.78	4.85	13.65	22.19	18.35	8.93	11.14	7.89	8.11	9.07	5.65
	ISE [58]	CVPR-22	20.01	18.93	8.54	14.22	24.62	21.74	16.12	16.99	13.24	10.83	13.66	10.71
USL-VI-ReID	H2H [59]	TIP-21	30.15	29.40	-	-	-	-	23.81	18.87	-	-	-	-
	OTLA [19]	ECCV-22	29.98	27.13	-	29.8	38.8	-	32.9	29.7	-	32.1	28.6	-
	ADCA [34]	MM-22	45.51	42.73	28.29	50.60	59.11	55.17	67.2	64.05	52.67	68.48	63.81	49.62
	DOTLA [16]	MM-23	50.36	47.36	32.40	53.47	61.73	57.35	85.63	76.71	61.58	82.91	74.97	58.60
	MBCCM [35]	MM-23	53.14	48.16	32.41	55.21	61.98	57.13	83.79	77.87	65.04	82.82	76.74	61.73
	CHCR [60]	TCSVT-23	47.72	45.34	-	50.12	42.17	-	68.18	63.75	-	69.08	63.95	-
	TAA [17]	TIP-23	48.77	42.43	25.37	50.12	56.02	49.96	62.23	56.00	41.51	63.79	56.53	38.99
	PGMAL [18]	CVPR-23	57.27	51.78	34.96	56.23	62.72	58.13	69.48	65.41	-	69.85	65.17	-
	MIMR [61]	KBS-24	46.56	45.88	-	52.26	60.93	-	68.76	64.33	-	68.76	63.83	-
	IMSL [62]	T-CSVT-24	57.96	53.93	-	58.30	64.31	-	70.08	66.30	-	70.67	66.35	-
	BCGM [63]	MM-24	61.70	56.1	38.7	60.9	66.5	62.3	86.8	81.7	68.6	86.7	82.3	71.1
	MMM [64]	ECCV-24	61.60	57.9	-	64.4	70.4	-	89.70	80.50	-	85.80	77.0	-
	PCLHD [65]	NIPS-24	64.40	58.70	-	69.50	74.40	-	84.30	80.70	-	82.70	78.40	-
	PCAL [40]	TIFS-25	57.94	52.85	36.90	60.07	66.73	62.09	86.43	82.51	72.33	86.21	81.23	68.71
	RULN [66]	AAAI-25	61.81	58.92	45.01	67.04	73.08	69.42	88.75	82.14	68.75	82.41	75.73	-
	DLM [67]	TPAMI-25	62.15	58.42	43.70	67.31	72.86	68.89	87.55	82.83	71.93	86.84	81.94	68.96
	RoDE [68]	TIFS-25	62.88	57.91	43.04	64.53	70.42	66.04	88.77	78.98	67.99	85.78	78.43	62.34
	SALCR [69]	IJCV-25	64.44	60.44	45.19	67.17	72.88	68.73	90.58	83.87	70.76	88.69	82.66	66.89
	DMDL [70]	PR-26	65.90	61.86	47.53	70.66	75.45	71.66	90.63	85.33	73.79	90.30	85.04	72.00
	Ours	-	66.20	62.79	49.42	69.36	74.72	70.84	93.34	87.55	76.08	91.89	86.58	73.60

**Table 2:** Complexity Comparison between Typical Backbones and Our Method.

Mehod	Input Size	Backbone	Params (M)	FLOPs (G)	FPS
CNN	384 × 128	ResNet-50	25.6	4.1	79.8
Transformer	224 × 224	ViT-Base	86.5	17.6	26.2
My Method	224 × 224	ViT-Base	91.5	31.8	14.3

**Table 3:** Ablation studies on the SYSU-MM01 and RegDB. Rank at r accuracy (%), mAP (%) and mINP (%) are reported.

						SYSU-MM01						RegDB					
	Components					All Search			Indoor Search			Visible to Infrared			Infrared to Visible		
Index	Baseline	VRA	VRNL	UM	PA-CBAM	r1	mAP	mINP	r1	mAP	mINP	r1	mAP	mINP	r1	mAP	mINP
1	√					52.07	52.23	40.64	54.89	63.95	60.49	54.17	50.42	36.19	52.91	48.38	34.47
2	√				√	53.24	53.41	41.70	55.97	65.06	61.65	55.03	51.40	37.23	54.18	49.46	35.24
3	√			√		55.61	55.57	44.02	58.05	67.15	63.56	60.10	56.57	42.12	59.41	54.49	39.67
4	√		√			56.13	53.67	41.08	59.11	64.42	60.85	56.13	52.29	37.16	54.35	49.73	36.04
5	√	√				62.86	61.54	48.62	68.06	73.57	69.70	91.73	85.74	73.69	90.58	84.63	70.59
6	√	√	√			64.33	62.20	49.06	68.24	74.18	69.82	92.35	86.22	74.31	91.13	85.18	71.01
7	√	√	√	√		65.14	62.58	49.31	69.97	74.34	70.26	92.61	86.59	74.45	91.38	85.42	71.39
8	√	√	√	√	√	**66.20**	**62.79**	**49.42**	**69.36**	**74.72**	**70.84**	**93.34**	**87.55**	**76.08**	**91.89**	**86.58**	**73.60**

**Table 4:** Contributions of Each Module and CA Modality in the Proposed Framework.

Component	Core Design Objective	Key Challenges Solved	Core Contributions to USL-VI-ReID
CA Modality	Treat channel augmentation as an independent input modality instead of simple data augmentation	Severe visible–infrared modal gap; underutilization of channel enhancement information	1. Serves as a bridge modality between visible and infrared spectra to narrow the inherent feature distribution gap; 2. Provides complementary cross-spectral feature cues for all subsequent modules; 3. Enables three-modal adversarial learning (MAAL) to enhance cross-modal feature consistency
PA-CBAM	Extract fine-grained and discriminative pedestrian features via two-level attention mechanism	Insufficient feature granularity; weak local identity feature representation; heavy background noise interference	1. Realizes channel + multi-scale spatial dual attention refinement to capture local discriminative cues (e.g., clothing textures, body parts); 2. Suppresses background noise and enhances identity-related feature channels; 3. Provides high-quality fine-grained feature foundation for subsequent cross-modal alignment and neighbor learning
VRA (with MAAL)	Achieve precise cross-modal regional feature alignment and reduce modal discrepancies	Imperfect global/local cross-modal alignment; ineffective use of multimodal information; low cross-modal similarity calculation reliability	1. Integrates MAAL to implement three-modal adversarial learning for global feature distribution alignment; 2. Realizes regional-level cross-modal feature matching to make up for the deficiency of only global alignment in existing methods; 3. Optimizes cross-modal clustering similarity calculation and improves the accuracy of cross-modal label assignment
VRNL	Enhance the reliability of neighbor learning and stabilize pseudo-labels via multi-region spatial association	Unreliable cross/intra-modal neighbor matching; pseudo-label noise caused by residual modal differences; identity confusion from excessive cross-modal matching	1. Constructs multi-region spatial associated neighbor sets to improve the consistency of cross-modal neighbor learning; 2. Imposes parameter constraints to avoid excessive cross-modal matching and reduce identity confusion; 3. Filters noisy samples in neighbor sets and stabilizes pseudo-label quality for subsequent clustering
UM	Merge split clusters of the same identity and suppress clustering noise	Identity cluster fragmentation; large intra-class feature variance; noise interference in clustering process	1. Adopts alternating contrastive learning to merge split clusters of the same ID and reduce intra-modal feature variance; 2. Eliminates clustering noise and improves the robustness of identity feature representation; 3. Optimizes the final clustering result and further enhances the accuracy of cross-modal identity matching

## Data Availability

The data that support the findings of this study are available online. These datasets were derived from the following public resources: [SYSU-MM01, RegDB].
